# Serum Level of Transferrin Unique Peptide Is Decreased in Patients With Acute Ischemic Stroke

**DOI:** 10.3389/fneur.2021.619310

**Published:** 2021-02-05

**Authors:** Xizheng Hu, Yinghui Li, Peng Cheng, Anhua Wu, Guangyu Li

**Affiliations:** ^1^Department of Neurosurgery, The First Hospital of China Medical University, Shenyang, China; ^2^Department of Medical Genetics, School of Life Science, China Medical University, Shenyang, China

**Keywords:** acute ischemic stroke, multiple reaction monitoring, labeled reference peptide, transferrin unique peptide, albumin unique peptide

## Abstract

**Objectives:** Free irons are transported into brain tissues by transferrin and play an important role in neuronal/glial cell damage. Lower serum levels of transferrin have been found in patients with ischemic stroke, compared with healthy subjects. In present study, we investigated whether transferrin unique peptide (TF-UP) could be employed as a serum biomarker for brain tissue damage in acute ischemic stroke.

**Methods:** The venous blood samples of 94 ischemic stroke patients and 35 *brain tumor-stroke mimics (BT-SM)* patients were collected within the first 72 h (Median time 23.25, Interquartile range 60.75) of acute onset in the emergency room. Total TF-UP and total albumin unique peptide (Alb-UP) were identified with liquid chromatography/mass spectrometry (LC–MS/MS) and quantified by multiple reaction monitoring (MRM) method using labeled reference peptide (LRP) for further analysis.

**Results:** Median ratio of total TF-UP/LRP was 0.85 (Interquartile range, 0.21) in the *brain tumor-stroke mimics (BT-SM)* group, and 0.45 (0.14) in the ischemic stroke group; median Alb-UP/LRP ratio was 0.66 (0.16) in the *BT-SM* group, and 0.55 (0.20) in the ischemic stroke group. The overall trend from low to high levels was statistically significant for TF-UP/LRP (*P* < 0.0001), but not for Alb-UP/LRP (*P* = 0.1667). According to the receiver operating characteristic (ROC) curve, the area under the curve (AUC) was 0.9565 and the optimal cutoff value of serum TF-UP was 0.6317, which yielded a sensitivity of 91.49% and a specificity of 88.57%. The odds ratio (95% confidence intervals) of serum TF-UP/LRP was 83.31 (23.43, 296.22, *P* < 0.0001).

**Conclusions:** Serum TF-UP/LRP level is decreased in patients with acute ischemic stroke in comparison with brain tumor, and it may serve as a serum biomarker for the neuronal/glial cell damage in cerebral infarction.

## Introduction

Oxidative stress plays an important role in neuronal injuries and free iron is responsible for oxidative damage in the infarcted brain tissues (1–4). Serum ferritin level at admission could be used as an independent predictor of short-term mortality and long-term functional outcome in neurological critically ill patients (5). Increased serum ferritin levels at admission have been also associated with poor outcome in ischemic stroke (6). Higher iron status has been reported to be associated with increased stroke risk, especially cardioembolic stroke (7). Transferrin (TF) is a plasma glycoprotein that reversibly binds to free iron in the blood and transport the free irons into cells. TF loaded with irons bind to the TF receptors (TFR) on the cell membranes and get into the cells by receptor-mediated endocytosis (3, 8). Systemic iron, TF, transferrin saturation (TSAT) and hepcidin have important clinical implications in acute ischemic stroke (AIS)-induced cell death (9). TF is the main protein regulating Fe hemostasis (8). Gillum et al. reported a significant association of TF saturation with risk of incident stroke in white women aged 45–74 (10). Altamura et al. found that serum TF level correlated inversely with the ischemic lesion volume in the brain and might play a protective role in the early phases of ischemic stroke (1). In addition, Ding et al. reported that the expression of ferritin and TFR was increased in the ischemic cerebral cortex and hippocampus, but the expression of ferroprotein 1, an iron-export protein, and hepcidin, the key regulator of ferroprotein 1, was significantly decreased in infarcted brain tissues (11). In a case-control study, lower level of TF and higher level of ferritin were found in the serum of 42 stroke patients, compared with 62 healthy controls (2). All these observations suggest that TF is involved in the brain tissue infarction. However, to our knowledge, the correlation between serum TF level and the probability of acute ischemic stroke has not been studied before.

In the past two decades, TFR has been identified as the target of drug delivery system, which moves across the blood brain barrier via TFR-mediated endocytosis (8, 12–14). Conjugated brain-derived neurotrophic factor (BDNF) to a monoclonal antibody against TFR demonstrated nearly 70% neuroprotection in focal brain ischemia after intravenous injection of conjugated BDNF (15–17). Furthermore, TF-coupled liposomes were used to deliver vascular endothelial growth factor (VEGF) into ischemic brain tissues, and significant improvement in the neurological function was observed (18). Based on these observations, it could be inferred that serum level of TF may be decreased in a TFR-specific manner in patients with acute ischemic stroke. It has been reported that the albumin (Alb) level decreased in AIS. However, undernutrition in the early stage of ischemic stroke may lead to a reduced Alb level in the serum, which may cause a unspecific change of Alb (19, 20). Moreover, Yoo et al. found that baseline undernutrition independently predicted 1-week undernutrition and more complications after the ischemic stroke (21).

The protein concentration is usually measured by the enzyme-linked immunosorbent assay (ELISA), and unique peptides of a target protein can be detected by triple-quadrupole mass spectrometry (MS). A potential advantage of mass spectrometry is to recognize all possible variations of a target protein, including both integrate and fragmental proteins. In 2015, Lian et al. developed a labeled reference peptide (LRP) method which employed a single labeled peptide as a reference standard for all targeted peptides and measured peptides based on their peak areas for detected MRM transitions (22). Therefore, unique peptides from all possible variants of TF protein or Alb protein in the serum could be identified and quantified by MRM-LRP, as a potential clinical laboratory test.

In the above studies, serum TF and Alb level were applied to screen high-risk population for stroke, whereas the sensitivities and specificities of TF and Alb levels as diagnostic tests have not been tested yet. A certain proportion of patients who are diagnosed with acute stroke symptoms will eventually find out that the cause is not an acute stroke, especially if the diagnosis of emergency imaging is not clear. A systematic review identified the most common stroke/TIA mimics across 29 cohorts (mostly in Europe and North America) and showed that brain tumor patients accounted for about 8% of the stroke/TIA mimics patients (23). Based on these observations, we employed brain tumor as a control. In the present study, brain tumors patients without AIS, were included as the BT-SM group, instead of healthy subjects to evaluate the clinical diagnostic efficiency.

## Materials and Methods

### Patients

A total of 129 patients were consecutively admitted to the department of neurology at the first hospital of China Medical University from October 2014 to December 2014, in Shenyang, China. Ninety-four patients with ischemic stroke within the first 72 h of acute onset were included as the AIS group, and 35 patients with glioblastoma, ependymoma or meningioma were enrolled as the BT-SM group (the control group). Demographic characteristics and history of risk factors, including gender, age, diabetes mellitus (DM), hypertension and previous ischemic heart diseases (IHD), were listed in Table 1. Routine blood tests, biochemical tests, and brain CT scans were performed at admission. The study was approved by The Ethics Committee for Medical Research at the First Hospital of China Medical University and performed in accordance with the ethical standards laid down in the 1964 Declaration of Helsinki and its later amendments.

**Table 1:** Demographic characteristics and mass spectrometry measurements of the study population.

**Variables**	**Total (*n* = 129)**	**BT-SM (*n* = 35)**	**Ischemic stroke (*n* = 94)**	***P* value**
Age, mean (SD)	57.97 (14.96)	46.71 (15.46)	62.16 (12.46)	<0.0001
**Gender, %**				
Male	59.69	60.00	59.57	0.5947
Female	40.31	40.00	40.43	0.5650
DM, %	12.40	2.86	15.96	0.0348
Hypertension, %	30.23	5.71	39.36	<0.0001
Previous IHD, %	10.85	2.86	13.83	0.0630
TF-UP/LRP, median (IQR)	0.50 (0.28)	0.85 (0.21)	0.45 (0.14)	<0.0001
Alb-UP/LRP, median (IQR)	0.58 (0.20)	0.66 (0.16)	0.55 (0.20)	<0.0001
TF-UP/Alb-UP, median (IQR)	0.87 (0.39)	1.25 (0.31)	0.79 (0.22)	<0.0001

*A Student's t test, Fisher's exact test (one-tail), or Wilcoxon rank sum test was used*.

*Results are expressed as percentages or as mean (Standard Deviation, SD) and medians (Interquartile Range, IQR); DM, diabetes mellitus; IHD, ischemic heart diseases; TF-UP, transferrin unique peptide; LRP, labeled reference peptide; Alb-UP, albumin unique peptide*.

### Reagents

Chloral hydrate was purchased from KeLong Chemical (Chengdu, China). Deionized water was purified by a Millipore Advantage A10 system (Millipore, Bedford, MA, USA). An amount of 90% ethanol (HPLC grade) was purchased from Thermo Fisher Scientific (Rockford, IL, USA). Sequencing grade trypsin was obtained from Promega (Madison, WI, USA). Ammonium formate was purchased from Thermo Fisher Scientific (Rockford, IL, USA). HPLC grade acetonitrile (CH3CN) was obtained from Thermo Fisher Scientific (Rockford, IL, USA). Isotopically labeled peptide with 99.8% purity: APGLTQALNTK (13C6, 15N2), and unlabeled peptide with the same sequence with 99.0% purity: APGLTQALNTK, were purchased from GenScript (Nanjing, China).

### Sample Preparation and MS Measurement of the Study Population

Venous blood was collected from all participants within 1 h after admission in vacutainer tubes and quickly centrifuged to avoid hemolysis. Then the serum samples were kept at −80°C and analyzed by investigators blinded to the clinical outcomes and neuroimaging findings. Right before the digestion, clinical plasma samples were taken from a −80°C freezer and thawed on ice. Protein concentration of each sample was determined with BCA assay. Briefly, 5 μL of each sample were transferred to a protein low-binding tube and trifluoroethanol (TFE)/100 mM ammonium bicarbonate (1:1, v:v) were added to make the final concentration of each tube 20 μg/μL. Samples containing 40 μg protein were aliquot and 13 μL TFE/ammonium bicarbonate (1:1, v:v) was added. Samples were then reduced with dithiothreitol (10 mM) at 50°C for 15 min followed by alkylation with iodoacetamide (20 mM) in the dark at room temperature for 15 min. The buffer was adjusted to 65 μL and tryptic digestion performed at 37°C overnight on a shaker. After digestion, the samples were dried in a speed vac concentrator (Thermo SAVANT SPD1010, ThermoSavant, Holbrook, NY, USA). The digests were resuspended in 0.1% formic acid. Each sample was spiked with 1.0 ng/μL APGLTQALNTK^*^ before liquid chromatography/mass spectrometry (LC–MS/MS) analysis.

One ng of the reference peptide (APGLTQALNTK^*^) with 13C and 15N labeling at the C-terminal lysine was added to each injection. The peak area of labeled reference peptide (LRP) was measured in each experiment to show the reliability and reproducibility of mass spectrometry analysis. Additionally, 3 unique peptides (DGAGDVAFVK, HSTIFENLANK, and EFQLFSSPHGK) for TF and another 3 unique peptides (EFNAETFTFHADICTLSEK, ALVLIAFAQYLQQCPFEDHVK, and NECFLQHK) for Alb protein were applied to cover integral and all possible fragmental TF proteins (Supplementary Table 1). The detection of each unique peptide by MRM was independent, and thus the mean value of the peak areas of 3 unique peptides was used to represent total TF-UP or total Alb-UP in each patient. The serum level of total TF-UP or Alb-UP was defined as the peak area ratio of total TF-UP to its corresponding LRP (TF-UP/LRP) or Alb-UP to LRP (TF-UP/LRP). The peak area ratio of total TF-UP to total Alb-UP (TF-UP/Alb-UP) in each sample was used to represent the relative abundance of TF.

### Selection of Unique Peptides, Reference Peptides, and Q1/Q3 Transition

Three transferrin unique peptides (DGAGDVAFVK, HSTIFENLANK, and EFQLFSSPHGK), three albumin unique peptides (EFNAETFTFHADICTLSEK, ALVLIAFAQYLQQCPFEDHVK, and NECFLQHK), and one labeled reference peptide (APGLTQALNTK^*^) were chosen due to their strong MRM transition signals, suitable elution time, and low variability in peak area. Three MS1/MS2 transition ion pairs were selected to verify a unique peptide. Multiple injections of the digests were performed to match relative intensities of target ion pair transition signals with those of MRM transition signals observed previously in ion trap MS/MS spectra obtained from the database. Reproducibility of transition signals between different runs was also examined to eliminate the false positive rates of proteins. Collision energy and delustering potential were calculated based on the empirical equation recommended by Skyline (24). The parameters of transition ion pairs for transferrin unique peptides, albumin unique peptides, reference peptide and labeled reference peptide were provided in Supplementary Table 1. MRM acquisition methods were performed with fragment ion-specific tuned Collision Energy (CE) voltages and scheduled retention time. The dynamic MRM option was used for all data acquisition with a target cycle time of 1 s, with a minimum and maximum dwell time of 14 and 333 ms (75 maximum concurrent MRMs) and a 300-s MRM detection window for MRM transitions. LC-MRM- MS analyses were performed as previously described (22).

### Statistical Analysis

Continuous variables were reported as mean (standard deviation, S.D.) or median (interquartile range, IQR) depending on their normal distribution, whereas categorical variables were expressed as ratios (percentage). Proportions were compared using Fisher's exact test and means or medians between groups compared by student's *t* test or Wilcoxon rank sum test as appropriate. The independent association between TF-UP level and the presence of ischemic stroke was estimated by a binary logistic regression analysis and the confounding factors were identified with stratification. Cochran Armitage trend test was used to detect any correlation between unique peptide levels (low, medium, and high) and the probability of ischemic stroke within each stratum. The independent effects of serum total TF-UP/LRP, Alb-UP/LRP, and TF-UP/Alb-UP on the probability of ischemic stroke were analyzed by multivariate logistic regression, which allows adjustment for confounding factors, such as age, diabetes mellitus, hypertension, and previous ischemic heart diseases. The results were demonstrated as adjusted odds ratios (ORs) and the corresponding 95% confidence intervals (CIs). Receiver operating characteristic (ROC) curve and its area under the curve (AUC) were used to evaluate the accuracy of serum TF-UP/LRP, Alb-UP/LRP, and TF-UP/Alb-UP ratio as a diagnostic test of AIS. JMP 10 (SAS Institute Inc., Cary, NC, USA) software was used for all statistical analysis. *P* values of <0.05 were considered significant. PASS15.0 software was used for calculating the statistical power of ROC curve. Calculation results showed that the AUC statistical power of TF-UP/LRP, Alb-UP/LRP, and TF-UP/Alb-UP are >0.99999, 0.99972, >0.99999, respectively, which are all >0.8, indicating that the statistical power is qualified for further analysis.

## Results

### Demographic Characteristics of the Study Population

This is a hospital-based observational cohort study of 94 patients with ischemic stroke within the first 72 h (Median time 23.25, IQR 60.75) of acute onset and 35 patients with brain tumors as the BT-SM group. Demographic characteristics and relevant mass spectrometry measurements of the study population are shown in Table 1. The mean age of these patients was 57.97 ± 14.96 years and 59.69% were men in the cohort. Patients in different groups (ischemic stroke, and BT-SM group) were similar in terms of gender, but patients with ischemic stroke (62.16 ± 12.46 years) were older than the BT-SM group (46.71 ± 15.46 years). The prevalence of diabetes mellitus or hypertension was significantly higher in the ischemic stroke group (Table 1).

### MS Measurements of the Study Population

Because the distribution of values for TF-UP/LRP, Alb-UP/LRP, and TF-UP/Alb-UP were skewed toward the lower end, the medians and interquartile ranges were used to describe the variabilities within samples. As shown in Table 1, the median serum TF-UP/LRP was 0.50 (IQR, 0.28) in total samples, 0.85 (0.21) in the BT-SM group, and 0.45 (0.14) in the AIS group, with a *P* value of <0.0001. The serum level of Alb-UP was lower in the ischemic stroke group [0.55 (0.20)], compared with that in the BT-SM group [0.66 (0.16), *P* < 0.0001]. The medians of Alb-UP in these two groups were no significant difference. The median of TF-UP/Alb-UP was also decreased in the ischemic stroke group in a statistically significant manner by single variate analysis (*P* < 0.0001).

### Stratification of the Study Population

As shown in Table 2, the numbers of patients with acute ischemic stroke across categories of TF-UP serum level (low level, 0– <0.4; medium level, 0.4– <0.8; high level, 0.8–1.2) were stratified by gender, age, diabetes mellitus, hypertension and previous ischemic heart diseases. Similar trends for the presence of ischemic stroke were found in men and women from low to high level of TF-UP. In young patients (0–29 years) and old patients (60–89 years), the trends were not statistically significant (*P* = 0.0504, and 0.0671, respectively). This suggests that age was a confounding factor for the effect of TF-UP on ischemic stroke. In patients without diabetes mellitus, or hypertension, or previous ischemic heart diseases, TF-UP level was significantly associated with the presence of ischemic stroke. Additionally, Diabetes mellitus, hypertension and previous ischemic heart diseases were confounding factors, because no statistically significant correlation between TF-UP serum levels and probability of acute ischemic stroke was noted in strata with those disorders. As shown in Supplementary Table 2, no statistically significant trend was observed across categories of Alb-UP serum levels (low level, 0– <0.4; medium level, 0.4– <0.8; high level, 0.8–1.2), stratified by gender, age, diabetes mellitus, hypertension, and previous ischemic heart diseases. All together, these results indicated that the difference among the Alb-UP levels in each stratum was not statistically significant due to smaller sample sizes after stratification.

**Table 2:** Case numbers of patients across categories of serum TF-UP level, stratified by gender, age, diabetes mellitus, hypertension, and previous ischemic heart diseases.

**Demographic characteristics**	**No. of patients**		**Serum TF-UP/LRP level**						***P* value**
	**I.S**.	**BT-SM**	**Low**		**Medium**		**High**		
			**I.S**.	**BT-SM**	**I.S**.	**BT-SM**	**I.S**.	**BT-SM**	
Overall	94	35	25	0	69	13	0	22	<0.0001
**Gender**									
Male	56	21	18	0	38	7	0	14	<0.0001
Female	38	14	7	0	31	6	0	8	<0.0001
**Age**									
0–29	1	4	0	0	0	1	0	3	0.0504
30–59	42	25	5	0	37	6	0	19	<0.0001
60–89	51	6	19	0	32	6	0	0	<0.0671
**Diabetes mellitus**									
Yes	15	1	4	0	11	1	0	0	0.5510
No	79	34	21	0	58	12	0	22	<0.0001
**Hypertension**									
Yes	37	2	9	0	28	2	0	0	0.4265
No	57	33	16	0	41	11	0	22	<0.0001
**Previous IHD**									
Yes	13	1	5	0	8	1	0	0	0.4392
No	81	34	20	0	61	12	0	22	<0.0001

*A Cochran-Armitage trend test was used. Results are expressed as percentages or as means (SD); I.S., ischemic stroke; BT-SM, brain tumor-stroke mimics; TF-UP, transferrin unique peptide; LRP, labeled reference peptide; IHD, ischemic heart diseases; Serum TF-UP/LRP level: low, 0– <0.4; medium, 0.4– <0.8; high, 0.8–1.2*.

### Multivariate Logistic Regression Analysis of TF-UP/LRP Ratio and the Probability of Acute Ischemic Stroke

The independent effect of serum TF-UP level on the probability of acute ischemic stroke was estimated by multivariate logistic regression, with adjustment for age, diabetes mellitus, hypertension, and previous ischemic heart diseases. Multivariate logistic regression analysis demonstrated a significant inverse relation between serum TF-UP level and the presence of ischemic stroke (*P* < 0.0001). In patients with hypertension, the probability of acute ischemic stroke was increased by 4.45-fold, with a 95% CI of 0.84–36.32 (Supplementary Table 3, logistic regression 1). Supplementary Table 3 also shows that odds ratios of age, diabetes mellitus, hypertension, and previous IHD in logistic regression 2 were similar to those in logistic regression 1. However, OR of diabetes mellitus was much higher in logistic regression 3 (10.93) than those in regression 1 and 2 (1.21 and 2.08, respectively), which indicated that the correlation between TF-UP/Alb-UP and the probability of acute ischemic stroke was dramatically confounded by the history of diabetes mellitus.

### Receiver Operating Characteristic Curve Analysis of Serum TF-UP/LRP Ratio

**Figure 1:**
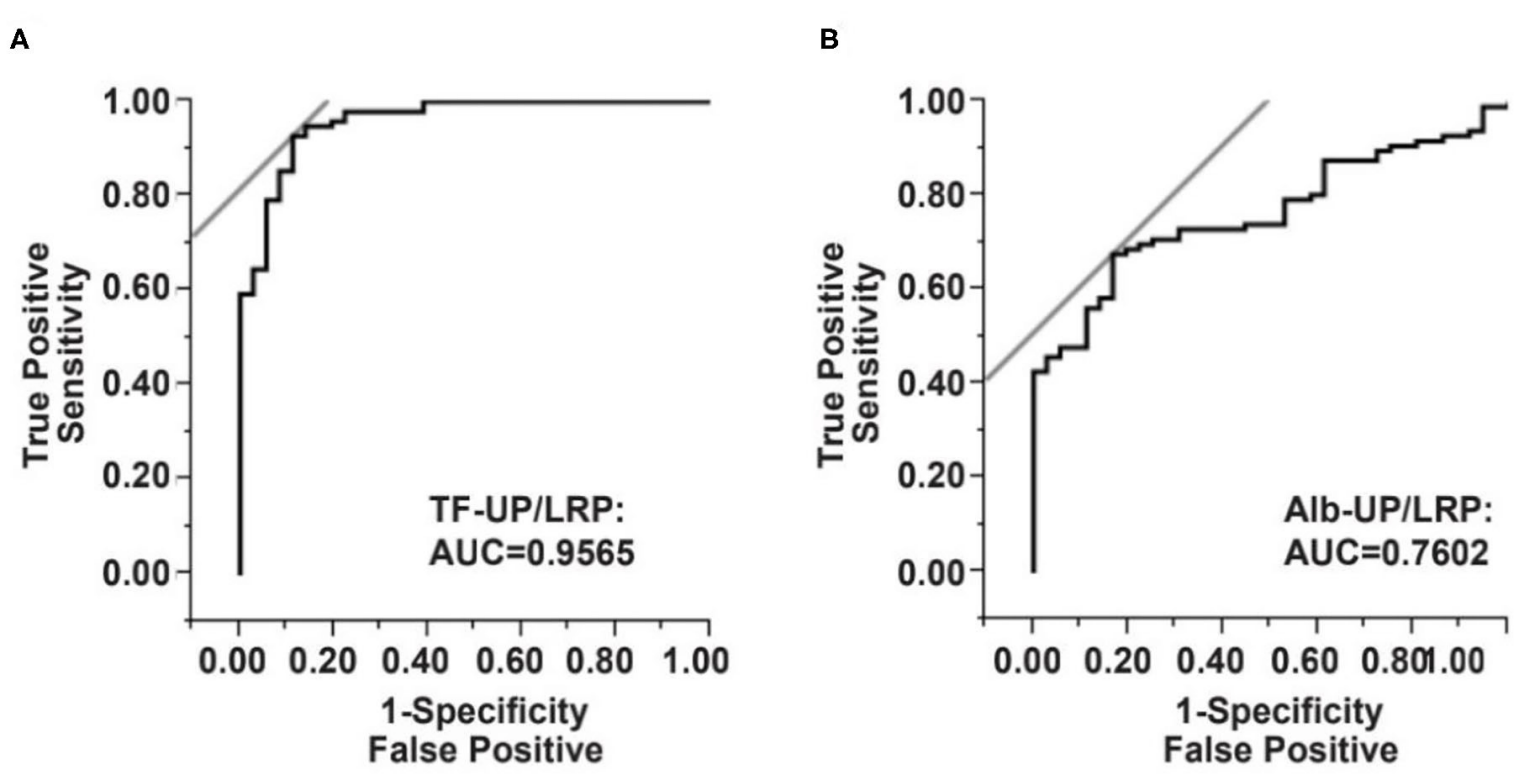
Serum TF-UP level and Alb-UP level as serum biomarkers for acute ischemic stroke, compared with brain tumor-stroke mimics (BT-SM) patients. Receiver operator characteristic (ROC) curve demonstrates sensitivity as a function of 1-specificity to confirm the presence of ischemic stroke by TF-UP/LRP ratio **(A)** or Alb-UP/LRP ratio **(B)**. TF-UP, transferrin unique peptide; LRP, labeled reference peptide; Alb-UP, albumin unique peptide; AUC, area under the curve.

The diagnostic efficiencies of TF-UP/LRP, Alb-UP/LRP, and TF-UP/Alb-UP were shown in Figure 1 and Table 3. Based on the ROC curve, the optimal cutoff value of TF-UP/LRP ratio as a biomarker for the presence of AIS was projected to be 0.6317 and the area under the curve (AUC) was 0.9565, which yielded a sensitivity of 91.49% and a specificity of 88.57% (Figure 1A and Table 3). Compared with the AUC of Alb-UP/LRP (0.7602) and TF-UP/Alb-UP (0.8793), TF-UP/LRP has better discriminatory ability. The cutoff value of Alb-UP level was 0.5963, with a sensitivity of 67.02% and a specificity of 82.86% (Figure 1B and Table 3). The diagnostic index was 1.8006 for TF-UP/LRP, 1.4988 for Alb-UP/LRP, and 1.6617 for TF-UP/Alb-UP, respectively (Table 3).

**Table 3:** Evaluation of TF-UP/LRP, Alb-UP/LRP and TF-UP/Alb-UP as serum biomarker for acute ischemic stroke.

**Variables**	**TF-UP/LRP**	**Alb-UP/LRP**	**TF-UP/Alb-UP**
AUC	0.9565	0.7602	0.8793
Sensitivity	0.9149	0.6702	0.8617
Specificity	0.8857	0.8286	0.8000
Diagnostic index	1.8006	1.4988	1.6617
Cut-off value	0.6317	0.5963	1.0423
OR	83.31	9.82	24.92
95% CI	23.43, 296.22	3.69, 26.13	9.04, 68.73
*P* value	<0.0001	<0.0001	<0.0001

*A Fisher's exact test (1-tail); OR, odds ratios; CI, confidence interval; TF-UP, transferrin unique peptide; LRP, labeled reference peptide; Alb-UP, albumin unique peptide; DI, diagnostic index; AUC, area under the curve*.

## Discussion

AIS is a common neurological disease. The rapid identification of these patients in clinics is essential for further proper treatment. In the present study, we observed an inverse correlation between serum TF-UP level and the presence of AIS, and found that a decrease in the serum TF-UP/LRP ratio was related to an increase in the positive rate of AIS, adjusting for age, DM, hypertension and previous IHD. The cutoff value of serum TF-UP/LRP ratio as a diagnostic biomarker was 0.6317 with a sensitivity of 91.49% and a specificity of 88.57%. The OR of TF-UP test was 83.31 and its 95% CI was 23.43–296.22. Therefore, compared with the BT-SM group, serum TF-UP level may serve as a reliable biomarker for the identification of neuronal/glial cell disruption in AIS. The test could return results within half an hour. The rapid detection of this serum biomarker will help the identification of brain tumor patients with similar symptoms like acute ischemic patients.

### Sensitivity of Alb-UP/LRP Ratio Is Low

The change in serum TF-UP level was statistically and clinically significant in patients with ischemic stroke, compared with the BT-SM group (Table 1). We found that age, diabetes mellitus, hypertension, and previous IHD were confounding factors of the TF-UP effect on the probability of AIS (Table 2 and Supplementary Table 3). To the contrast, the decrease in the serum Alb-UP level was statistically, but not clinically, significant (Table 1 and Supplementary Table 2). In each stratum, the sample size was decreased, and the *P*-value was increased (Supplementary Table 2). These results were consistent with previous studies, which showed that the change in Alb-UP level was mainly due to non-specific reasons like undernutrition (21, 25, 26). Additionally, in clinical settings, albumin protein may be systemically administered to correct the malnourishment (19). As a result, the sensitivity of Alb-UP/LRP was only 67.02%, much lower than 91.49% for TF-UP/LRP or 86.17% for TF-UP/Alb-UP in Table 3. Therefore, Alb-UP level is not a sensitive biomarker for the neural damage in acute ischemic stroke.

### Specificity of TF-UP/Alb-UP Ratio Is Low

The specificity of TF-UP/Alb-UP was only 0.8000, lower than that of TF-UP/LRP (0.8857) or Alb-UP/LRP (0.8286) (Table 3). Here, LRP is an external control peptide for quality control and quantification purpose. Alb-UP may serve as an internal control for the comparison of TF-UP levels of serum samples between the ischemic stroke group and the BT-SM group. However, the effect of TF-UP/Alb-UP was dramatically confounded by diabetes mellitus, with an OR of 10.93, much >1.21 for TF-UP (Supplementary Table 3). The OR of diabetes mellitus was even higher than that of hypertension (6.12) (Supplementary Table 3, logistic regression 3). This is not consistent with clinical findings, and may be an artifact. The diagnostic specificity of TF-UP/Alb-UP would be low in patients with diabetes mellitus. Further study is needed to clarify this issue.

### Limitations and Future Studies

Serum TF-UP/LRP ratio is a more sensitive and more specific biomarker for neural damage in acute ischemic stroke than Alb-UP/LRP ratio and TF-UP/Alb-UP ratio, in comparison with brain tumors. A certain proportion of patients who are presented with acute stroke symptoms will eventually find out that the cause is not an acute stroke, especially if the diagnosis of emergency imaging is not clear. A systematic review identified the most common stroke/TIA mimics across 29 cohorts (23). Brain tumor patients accounted for about 8% of these patients. The present study was aimed at quickly distinguish between brain tumor and AIS patients. Because of the limited collection time, our study has fewer patients in the BT-SM group, which may reflect the cohort characteristics of our hospital-based observational study. In the future, we will increase the sample size to explore whether transferrin is a sensitive biomarker for detecting AIS. In addition, healthy controls and more stroke mimic patients will be included to extend the application scope of TF-UP. Moreover, biomarkers that could distinguish between AIS and mimics at very early time points after AIS onset will help us to choose the proper treatment strategies. In the further study, it's worth exploring whether the level of TF-UP decreases in a time-dependent manner after AIS onset. This will give a clue of whether TF-UP levels reduce in a time-dependent manner post-stroke onset. A panel of multiple unique peptides, such as apolipoprotein A1 unique peptide (APOA1-UP) and glial fibrillary acidic protein unique peptide (GFAP-UP), can be included in a single diagnostic or prognostic test of acute ischemic stroke to further increase the sensitivity and the specificity. It has been reported that patients with high serum transferrin are at higher risk of type 2 diabetes (27). A strong positive correlation between transferrin and blood glucose level also has been confirmed (28). Additionally, a study showed that TF was positively correlated with abnormal glucose metabolism (29). An increase in blood sugar will lead to an increase in TF levels. These results may explain the influence of diabetic patients in this study. The present data have showed that serum TF-UP/LRP level in patients with acute ischemic stroke was lower than those in patients with brain tumors. It is necessary to include more stroke mimic patients (such as migraine, etc.) in future studies to determine whether TF-UP could identify AIS in a real-world population.

## Data Availability Statement

The original contributions presented in the study are included in the article/Supplementary Material, further inquiries can be directed to the corresponding author/s.

## Ethics Statement

The studies involving human participants were reviewed and approved by Ethics approval was granted by The Ethics Committee for Medical Research at the First Affiliated Hospital of China Medical University (Shenyang, China). This study has therefore been performed in accordance with the ethical standards laid down in the 1964 Declaration of Helsinki and its later amendments. The patients/participants provided their written informed consent to participate in this study.

## Author Contributions

XH, GL, and AW designed and directed the study. XH and GL carried out the clinical trial procedures including patient data collection and trial coordination. XH, PC, and YL contributed to the data analysis and to the writing of the manuscript. All authors contributed to the article and approved the submitted version.

## Conflict of Interest

The authors declare that the research was conducted in the absence of any commercial or financial relationships that could be construed as a potential conflict of interest.

## References

[1] Altamura C Squitti R Pasqualetti P Gaudino C Palazzo P Tibuzzi F. Ceruloplasmin/Transferrin system is related to clinical status in acute stroke. Stroke. (2009) 40:1282–8. 10.1161/STROKEAHA.108.536714 19228837

[2] Molina JA Jimenez-Jimenez FJ Ayuso-Peralta L Cabrera-Valdivia F Perez-Sempere A Egido JA. Peripheral iron metabolism in patients with focal cerebral ischemia. Eur J Neurol. (1995) 2:107–9. 10.1111/j.1468-1331.1995.tb00101.x 24283609

[3] Slomka A Switonska M Zekanowska E. Hepcidin levels are increased in patients with acute ischemic stroke: preliminary report. J Stroke Cerebrovasc Dis. (2015) 24:1570–6. 10.1016/j.jstrokecerebrovasdis.2015.03.031 25881778

[4] Sorond FA Ratan RR. Ironing-out mechanisms of neuronal injury under hypoxic-ischemic conditions and potential role of iron chelators as neuroprotective agents. Antioxid Redox Signal. (2000) 2:421–36. 10.1089/15230860050192206 11229356

[5] Xie L Peng Y Huang K Wu Y Wang S. Predictive value of iron parameters in neurocritically ill patients. Brain Behav. (2018) 8:e01163. 10.1002/brb3.1163 30451393 PMC6305919

[6] Millan M Sobrino T Castellanos M Nombela F Arenillas JF Riva E. Increased body iron stores are associated with poor outcome after thrombolytic treatment in acute stroke. Stroke. (2007) 38:90–5. 10.1161/01.STR.0000251798.25803.e0 17138950

[7] Gill D Monori G Tzoulaki I Dehghan A Iron status and risk of stroke. Stroke. (2018) 49:2815–21. 10.1161/STROKEAHA.118.022701 30571402 PMC6257507

[8] Hao J Bickel U. Transferrin receptor mediated brain uptake during ischemia and reperfusion. J Pharm Pharm Sci. (2013) 16:541–50. 10.18433/J3B303 24210062

[9] DeGregorio-Rocasolano N Marti-Sistac O Gasull T. Deciphering the iron side of stroke: neurodegeneration at the crossroads between iron dyshomeostasis, excitotoxicity, and ferroptosis. Front Neurosci. (2019) 13:85. 10.3389/fnins.2019.00085 30837827 PMC6389709

[10] Gillum RF Sempos CT Makuc DM Looker AC Chien CY Ingram DD. Serum transferrin saturation, stroke incidence, and mortality in women and men. The NHANES I Epidemiologic Followup Study. National Health and Nutrition Examination Survey. Am J Epidemiol. (1996) 144:59–68. 10.1093/oxfordjournals.aje.a008855 8659486

[11] Ding H Yan CZ Shi H Zhao YS Chang SY Yu P. Hepcidin is involved in iron regulation in the ischemic brain. PLoS ONE. (2011) 6:e25324. 10.1371/journal.pone.0025324 21957487 PMC3177902

[12] Omori N Maruyama K Jin G Li F Wang SJ Hamakawa Y. Targeting of post-ischemic cerebral endothelium in rat by liposomes bearing polyethylene glycol-coupled transferrin. Neurol Res. (2003) 25:275–9. 10.1179/016164103101201508 12739237

[13] Wang Z Zhao Y Jiang Y Lv W Wu L Wang B. Enhanced anti-ischemic stroke of ZL006 by T7-conjugated PEGylated liposomes drug delivery system. Sci Rep. (2015) 5:12651. 10.1038/srep12651 26219474 PMC4518266

[14] Yemisci M Caban S Gursoy-Ozdemir Y Lule S Novoa-Carballal R Riguera R. Systemically administered brain-targeted nanoparticles transport peptides across the blood-brain barrier and provide neuroprotection. J Cereb Blood Flow Metab. (2015) 35:469–75. 10.1038/jcbfm.2014.220 25492116 PMC4348388

[15] Zhang Y Pardridge WM. Neuroprotection in transient focal brain ischemia after delayed intravenous administration of brain-derived neurotrophic factor conjugated to a blood-brain barrier drug targeting system. Stroke. (2001) 32:1378–84. 10.1161/01.STR.32.6.1378 11387502

[16] Zhang Y Pardridge WM. Conjugation of brain-derived neurotrophic factor to a blood-brain barrier drug targeting system enables neuroprotection in regional brain ischemia following intravenous injection of the neurotrophin. Brain Res. (2001) 889:49–56. 10.1016/S0006-8993(00)03108-5 11166685

[17] Zhang Y Pardridge WM. Blood-brain barrier targeting of BDNF improves motor function in rats with middle cerebral artery occlusion. Brain Res. (2006) 1111:227–9. 10.1016/j.brainres.2006.07.005 16884698

[18] Zhao H Bao XJ Wang RZ Li GL Gao J Ma SH. Postacute ischemia vascular endothelial growth factor transfer by transferrin-targeted liposomes attenuates ischemic brain injury after experimental stroke in rats. Hum Gene Ther. (2011) 22:207–15. 10.1089/hum.2010.111 21128742

[19] Bielewicz J Kurzepa J Czekajska-Chehab E Kamieniak P Daniluk B Bartosik-Psujek H. Worse neurological state during acute ischemic stroke is associated with a decrease in serum albumin levels. J Mol Neurosci MN. (2016) 58:493–6. 10.1007/s12031-015-0705-4 26757706 PMC4829619

[20] Cojocaru IM Cojocaru M Sapira V Ionescu A Barlan S Tacu N. Could pro-BNP, uric acid, bilirubin, albumin and transferrin be used in making the distinction between stroke subtypes? Roman J Intern Med. (2013) 51:188–95. 10.1016/j.jns.2013.07.717 24620632

[21] Yoo SH Kim JS Kwon SU Yun SC Koh JY Kang DW. Undernutrition as a predictor of poor clinical outcomes in acute ischemic stroke patients. Arch Neurol. (2008) 65:39–43. 10.1001/archneurol.2007.12 18195138

[22] Lian T Qu D Zhao X Yu L Gao B. Identification of site-specific stroke biomarker candidates by laser capture microdissection and labeled reference peptide. Int J Mol Sci. (2015) 16:13427–41. 10.3390/ijms160613427 26110384 PMC4490502

[23] Gibson LM Whiteley W. The differential diagnosis of suspected stroke: a systematic review. J R Coll Phys Edinb. (2013) 43:114–8. 10.4997/JRCPE.2013.205 23734351

[24] MacLean B Tomazela DM Shulman N Chambers M Finney GL Frewen B. Skyline: an open source document editor for creating and analyzing targeted proteomics experiments. Bioinformatics. (2010) 26:966–8. 10.1093/bioinformatics/btq054 20147306 PMC2844992

[25] Makris K Koniari K Spanou L Gialouri E Evodia E Lelekis M. Prognostic significance of serum albumin level changes in acute ischemic stroke: the role of biological and analytical variation. Clin Chem Lab Med. (2016) 54:143–50. 10.1515/cclm-2015-0281 26124056

[26] Babu MS Kaul S Dadheech S Rajeshwar K Jyothy A Munshi A. Serum albumin levels in ischemic stroke and its subtypes: correlation with clinical outcome. Nutrition. (2013) 29:872–5. 10.1016/j.nut.2012.12.015 23422540

[27] Podmore C Meidtner K Schulze MB Scott RA Ramond A Butterworth AS. Association of multiple biomarkers of iron metabolism and type 2 diabetes: the EPIC-InterAct study. Diabetes Care. (2016) 39:572–81. 10.2337/dc15-0257 26861925 PMC5058436

[28] Huth C Beuerle S Zierer A Heier M Herder C Kaiser T. Biomarkers of iron metabolism are independently associated with impaired glucose metabolism and type 2 diabetes: the KORA F4 study. Eur J Endocrinol. (2015) 173:643–53. 10.1530/EJE-15-0631 26294793

[29] Fumeron F Pean F Driss F Balkau B Tichet J Marre M. Ferritin and transferrin are both predictive of the onset of hyperglycemia in men and women over 3 years: the data from an epidemiological study on the insulin resistance syndrome (DESIR) study. Diabetes Care. (2006) 29:2090–4. 10.2337/dc06-0093 16936158

