# Supplementary material for: Serum Level of Transferrin Unique Peptide Is Decreased in Patients With Acute Ischemic Stroke
Source: Front Neurol. 2021 Feb 5;12:619310. doi: 10.3389/fneur.2021.619310 (PMC7901928; doi:10.3389/fneur.2021.619310)
Supplement: Supplementary file 1 [file Table_4.DOCX]

***Supplementary Material***

**Supplementary Tables**

**Supplementary table 1.** MRM parameters for TF-UPs, Alb-UPs, and LRP.

**Supplementary table 2.**  Case numbers of patients across categories of serum Alb-UP level, stratified by gender, age, diabetes mellitus, hypertension and previous ischemic heart diseases.

**Supplementary table 3.** Adjusted odds ratios (OR) and 95% confidence intervals (CI) of age, diabetes mellitus, hypertension and previous IHD.

**Supplementary table 1. MRM parameters for TF-UPs, Alb-UPs, and LRP.**

| **Peptides** | **Sequences** | **Q1/Q3** | **Retention Time (min)** |
| --- | --- | --- | --- |
| **TF-UP1** | DGAGDVAFVK1 | 489.748/735.404 |  |
|  | DGAGDVAFVK2 | 489.748/563.355 | 9.77 |
|  | DGAGDVAFVK3 | 489.748/464.287 |  |
| **TF-UP2** | HSTIFENLANK1 | 425.223/688.362 | 13.9 |
|  | HSTIFENLANK2 | 425.223/559.320 |  |
|  | HSTIFENLANK3 | 425.223/332.193 |  |
| **TF-UP3** | EFQLFSSPHGK1 | 638.820/872.462 | 15.9 |
|  | EFQLFSSPHGK2 | 638.820/759.378 |  |
|  | EFQLFSSPHGK3 | 638.820/612.310 |  |
| **Alb-UP1** | EFNAETFTFHADICTLSEK1 | 1130.515/1421.673 |  |
|  | EFNAETFTFHADICTLSEK2 | 1130.515/1320.625 | 21.5 |
|  | EFNAETFTFHADICTLSEK3 | 1130.515/1173.557 |  |
| **Alb-UP2** | ALVLIAFAQYLQQCPFEDHVK1 | 1245.646/1400.663 |  |
|  | ALVLIAFAQYLQQCPFEDHVK2 | 1245.646/1031.462 | 24.5 |
|  | ALVLIAFAQYLQQCPFEDHVK3 | 1245.646/871.431 |  |
| **Alb-UP3** | NECFLQHK1 | 538.253/832.413 |  |
|  | NECFLQHK2 | 538.253/672.383 | 3.48 |
|  | NECFLQHK3 | 538.253/525.314 |  |
| **RP** | APGLTQALNTK1 | 557.317 / 945.536 |  |
|  | APGLTQALNTK2 | 557.317 / 775.431 | 10.9 |
|  | APGLTQALNTK3 | 557.317 / 674.383 |  |
| **LRP** | APGLTQALNTK*1 | 561.324 / 953.551 |  |
|  | APGLTQALNTK*2 | 561.324 / 783.445 | 10.9 |
|  | APGLTQALNTK*3 | 561.324 / 682.397 |  |

Note: MRM, multiple reaction monitoring; TF-UPs, transferrin unique peptides; Alb-UPs, albumin unique peptides; RP, reference peptide; LRP, labeled reference peptide.

**Supplementary table 2.**  Case numbers of patients across categories of serum Alb-UP level, stratified by gender, age, diabetes mellitus, hypertension and previous ischemic heart diseases.

| Demographic Characteristics | No. of Patients  I.S. BT-SM | Serum Alb-UP/LRP Level  Low Medium High ­  I.S. BT-SM I.S. BT-SM I.S. BT-SM | P value |
| --- | --- | --- | --- |
| **Overall** | 94 35 | 7 0 81 32 6 3 | <0.1667 |
| **Gender** |  |  |  |
| **Male** | 56 21 | 4 0 49 19 4 2 | <0.3004 |
| **Female** | 38 14 | 3 0 32 13 2 1 | <0.3574 |
| **Age** |  |  |  |
| **0-29** | 1 4 | 0 0 2 4 0 0 | ------------ |
| **30-59** | 42 25 | 3 0 37 22 1 3 | <0.0399 |
| **60-89** | 51 6 | 4 0 42 6 5 0 | <0.9089 |
| **Diabetes Mellitus** |  |  |  |
| **Yes** | 15 1 | 3 0 12 1 1 0 | 0.7897 |
| **No** | 79 34 | 4 0 69 31 5 3 | 0.2554 |
| **Hypertension** |  |  |  |
| **Yes** | 37 2 | 4 0 32 2 2 0 | 0.8482 |
| **No** | 57 33 | 3 0 49 30 4 3 | 0.3100 |
| **Previous IHD** |  |  |  |
| **Yes** | 13 1 | 3 0 9 1 1 0 | 0.7735 |
| **No** | 81 34 | 4 0 72 31 5 3 | 0.2475 |

Note: ^a^ Cochran-Armitage trend test was used.

Results are expressed as percentages or as means (SD); I.S., ischemic stroke; BT-SM, brain tumor-stroke mimics; Alb-UP, albumin unique peptide; LRP, labeled reference peptide; IHD, ischemic heart diseases; Serum Alb-UP/LRP level: low, 0-<0.4; medium, 0.4-<0.8; high, 0.8-1.2.

**Supplementary table 3.** Adjusted odds ratios (OR) and 95% confidence intervals (CI) of age, diabetes mellitus, hypertension and previous IHD.

| Logistic  Regression | Age | | | Diabetes Mellitus | | Hypertension | | Previous IHD |
| --- | --- | --- | --- | --- | --- | --- | --- | --- |
| 1 TF-UP/LRP  OR (95% CI) | | 1.03  (0.98,1.09) | 1.21  (0.14,26.63) | | 4.45  (0.84,36.32) | | 2.80 (0.23,77.02) | |
| 2 Alb-UP /LRP  OR (95% CI) | | 1.08 (1.04,1.13) | 2.08  (0.31,41.52) | | 8.10  (1.99,55.71) | | 2.03  (0.25,45.05) | |
| 3 TF-UP/Alb-UP  OR (95% CI) | | 1.06 (1.01,1.11) | 10.93  (0.99,351.80) | | 6.12  (1.15,59.80) | | 1.15  (0.11,34.47) | |

Note: IHD, ischemic heart diseases; TF-UP, transferrin unique peptide; LRP, labeled reference peptide; Alb-UP, albumin unique peptide
